# Whole exome sequencing in an Indian family links Coats plus syndrome and dextrocardia with a homozygous novel *CTC1* and a rare *HES7* variation

**DOI:** 10.1186/s12881-015-0151-8

**Published:** 2015-02-10

**Authors:** Manjunath Netravathi, Renu Kumari, Saketh Kapoor, Pushkar Dakle, Manish Kumar Dwivedi, Sumitabho Deb Roy, Paritosh Pandey, Jitender Saini, Anil Ramakrishna, Devaraddi Navalli, Parthasarathy Satishchandra, Pramod Kumar Pal, Arun Kumar, Mohammed Faruq

**Affiliations:** Department of Neurology, National Institute of mental health & Neurosciences (NIMHANS), Bangalore, 560029 India; Genomics and Molecular Medicine, CSIR-Institute of Genomics and Integrative Biology, Mall Road, New Delhi, India; Department of Molecular Reproduction, Development and Genetics, Indian Institute of Science, Bangalore, 560012 India; Proteomics and Structural Biology Unit, CSIR-Institute of Genomics and Integrative Biology, Mall Road, New Delhi, India; Department of Neurosurgery, National Institute of mental health & Neurosciences (NIMHANS), Bangalore, 560029 India; Department of Neuroimaging & Interventional Neuroradiology, National Institute of mental health & Neurosciences (NIMHANS), Bangalore, 560034 India

**Keywords:** *CTC1*, Coats plus syndrome, CRMCC, Whole exome sequencing, Autosomal recessive disease, Dextrocardia, Notch signaling

## Abstract

**Background:**

Coats plus syndrome is an autosomal recessive, pleiotropic, multisystem disorder characterized by retinal telangiectasia and exudates, intracranial calcification with leukoencephalopathy and brain cysts, osteopenia with predisposition to fractures, bone marrow suppression, gastrointestinal bleeding and portal hypertension. It is caused by compound heterozygous mutations in the *CTC1* gene*.*

**Case presentation:**

We encountered a case of an eight-year old boy from an Indian family with manifestations of Coats plus syndrome along with an unusual occurrence of dextrocardia and situs inversus. Targeted resequencing of the *CTC1* gene as well as whole exome sequencing (WES) were conducted in this family to identify the causal variations. The identified candidate variations were screened in ethnicity matched healthy controls. The effect of *CTC1* variation on telomere length was assessed using Southern blot. A novel homozygous missense mutation c.1451A > C (p.H484P) in exon 9 of the *CTC1* gene and a rare 3′UTR known dbSNP variation (c.*556 T > C) in *HES7* were identified as the plausible candidates associated with this complex phenotype of Coats plus and dextrocardia. This *CTC1* variation was absent in the controls and we also observed a reduced telomere length in the affected individual’s DNA, suggesting its likely pathogenic nature. The reported p.H484P mutation is located in the N-terminal 700 amino acid regionthat is important for the binding of *CTC1* to ssDNA through its two OB domains. WES data also showed a rare homozygous missense variation in the *TEK* gene in the affected individual. Both *HES7* and *TEK* are targets of the Notch signaling pathway.

**Conclusions:**

This is the first report of a genetically confirmed case of Coats plus syndrome from India. By means of WES, the genetic variations in this family with unique and rare complex phenotype could be traced effectively. We speculate the important role of Notch signaling in this complex phenotypic presentation of Coats plus syndrome and dextrocardia. The present finding will be useful for genetic diagnosis and carrier detection in the family and for other patients with similar disease manifestations.

**Electronic supplementary material:**

The online version of this article (doi:10.1186/s12881-015-0151-8) contains supplementary material, which is available to authorized users.

## Background

Mutations in the genes involved in the telomere length maintenance complex have been reported to be associated with a variety of clinically heterogeneous multisystem disorders. This includes Cerebro-Retinal Microangiopathy with Calcifications and Cysts (CRMCC)/Coats plus syndrome (*CTC1* mutations), Dyskeratosis Congenita (DC/nail dysplasia, skin pigmentation defects and oral leukoplakia; associated with mutations in *DKC1*, *CTC1* and seven other genes), and two other DC variant diseases, Hoyeraal-Hreidarsson syndrome (bone-marrow failure, intrauterine growth retardation, microcephaly, immunodeficiency and cerebellar atrophy; associated with *RTEL1*, *DKC1*, *TERT* and *TINF2* mutations) and Revesz syndrome (aplastic anemia, retinopathy, intracranial calcifications and cerebellar hypoplasia; associated with *TINF2* mutations) [1-3]. In all of these diseases, some features of DC like sparse/gray hair, nail dystrophy and bone marrow failure appear as the overlapping phenotype. In addition, idiopathic pulmonary fibrosis, gastrointestinal bleeding, skeletal deformities and predisposition to certain forms of cancer have been described with some of the telomere biology related genes like *CTC1* [3]. Since its initial identification, more than 20 recessive mutations associated with CRMCC/Coats plus syndrome and DC phenotype have been described in the *CTC1* gene [1,2,4,5]. In earlier literature on this disease CRMCC has been used as an umbrella term to encompass two separate but similar diseases: Coats Plus syndrome and LCC. However, after identification of *CTC1* mutation with Coats plus syndrome, the two are considered non-allelic, genetically distinct disorders and Coats plus is the appropriate term than CRMCC to encompass Cerebroretinal microangiopathy lesions with other non-neurological features [6]. Overall Coats plus syndrome shows phenotypic overlap with Revesz syndrome (exudative retinopathy, intracranial calcification, bone marrow failure and shortened telomeres) as well as Labrune syndrome (LCC; leukoencephalopathy, intracranial calcifications and cysts) [2,7]. In a few cases of Coats plus, cardiac lesions and abnormalities like microangiopathy, atrial and ventricular septal defects have also been reported [4].

Previously described *CTC1* mutations have been found to occur in a compound heterozygous state with one protein truncating mutation always coupled to another missense variant [2,4,5]. It has also been reported that *CTC1* variations that cause protein truncation effects telomere length shortening more than the missense variation in cell line models, although conflicting results have been observed in patient sample based telomere length analysis [8].

We encountered a patient who manifested typical features of Coats plus syndrome and showed an unusual association of dextrocardia with situs inversus (reversed pattern of internal visceral organs on the left-right axis). Initial screening for *CTC1* mutations by targeted resequencing identified a homozygous missense variation. Owing to this being the first description of a homozygous missense variant in *CTC1* to occur along with Coats plus and the presence of dextrocardia, we extended our search for mutation(s) in other telomere biology and dextrocardia related genes in this family using whole exome sequencing.

## Case presentation

### Clinical description

**Figure 1:**
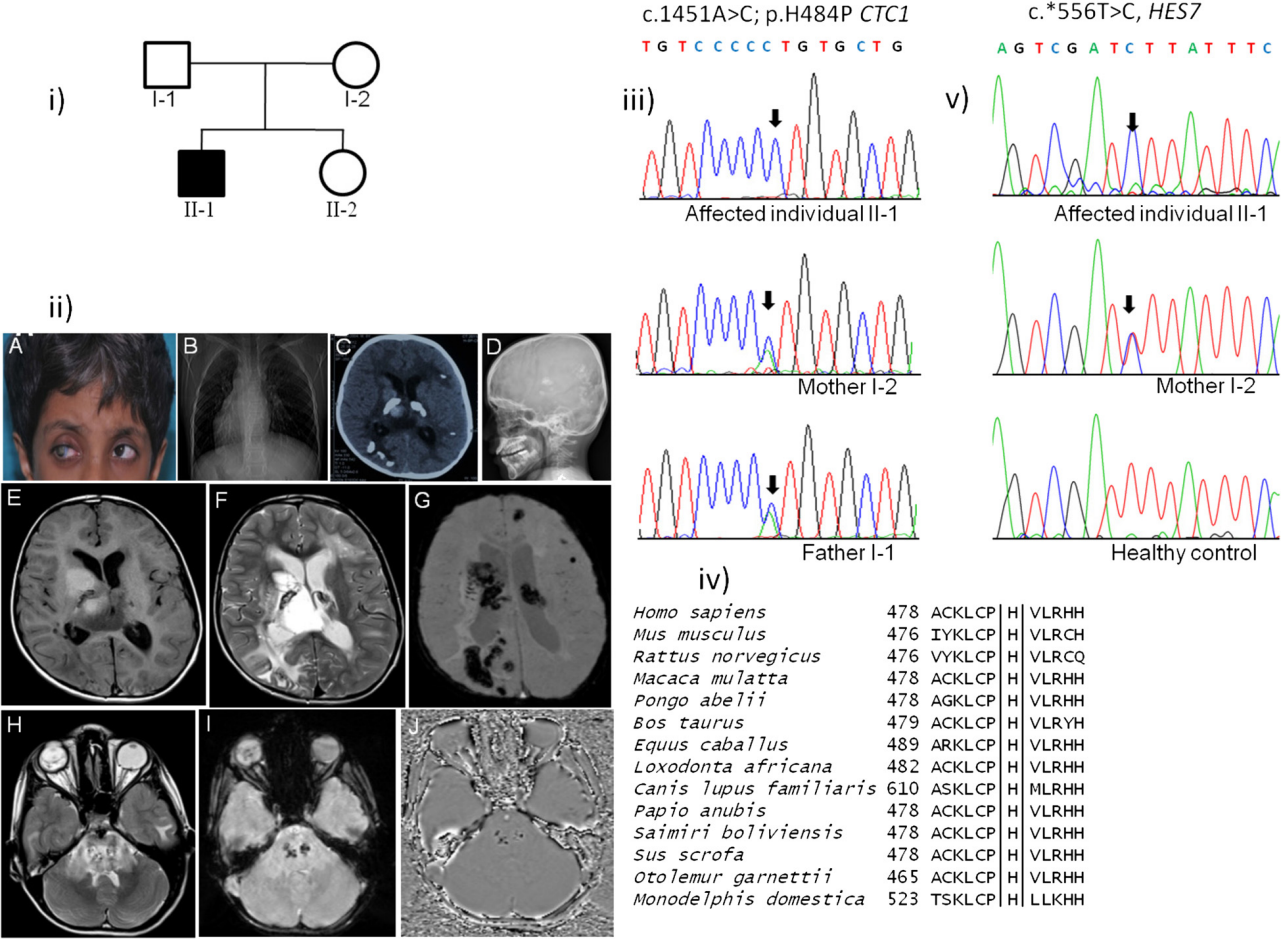
**Coats plus syndrome phenotype and mutation analysis of the** ***CTC1*** **and** ***HES7*** **in the family. i)** Pedigree diagram. **ii-A)** Photograph showing greying of the hair, right corneal opacity and leukocoria. **ii-B)** X-ray chest showing the presence of dextrocardia. **ii-C)** CT scan of the brain showing calcifications in bilateral thalamus, right parieto-occipital and left parietal regions. **ii-D)** An X-ray image of the lateral view of the skull showing the presence of intracranial calcifications. **ii-E)** Brain-MRI:Axial T1W image showing mixed intensity solid cystic lesion in the bilateral thalami. **ii-F)** Axial T2W image showing hyperintense lesion with few areas of T2 shortening and abundant perilesional edema. **ii-G)** Susceptibility weighted image (SWI) showing abundant susceptibility within the lesion as well as in the rest of the neuroparenchyma. **ii-H&I)** Similar lesions noted in the pons with abundant signal changes, susceptibility and edema. In addition, T2W axial image shows retinal detachment in the right eyeball. **ii-J)** SWI phase image showing hypointense signal intensity in the areas of blooming, which suggests the presence of calcification within the lesion. iii) Sequencing chromatograms from the affected individual (II-1) and both parents (I-1 and I-2). Note the nucleotide change A > C (arrow) in a homozygous state in the affected individual II-1 and in a heterozygous state in both parents. DNA from the unaffected individual II-2 was not available for genetic analysis. iv) Conservation of the H (histidine) residue across different species in the *CTC1* protein. v) sequencing chromatogram of *HES7* 3′UTR variation (T > C change, arrow marked) from affected individual (II-1), heterozygous parent(Mother) and a healthy control subject.

The propositus (II-1), an eight-year old boy of a non-consanguineous parentage (Figure 1, i) with mildly delayed developmental milestones, was referred to the National Institute of Mental Health and Neuro Sciences, Bangalore, for evaluation of gradually progressive left spastic hemiparesis of three months duration. At the age of 18 months, his eyes manifested retinal telangiectasia and exudates compatible with the diagnosis of the Coats Disease. He had received laser photocoagulation in the right eye, multiple sessions of injection of Avastin (Bevacizumab) and vitreous lavage. On physical examination, his vitals were stable. His neurological examination revealed mild left facial weakness with spasticity in the left upper and lower limb. His deep tendon reflexes were brisk bilaterally (right more than the left). Among extra-neurological features, he had convergent squint of right eye, premature graying of hair, two café-au-lait spots over the forearm (1×2 cm) and bilateral contracture of tendo Achilles (left more than right). Ophthalmologic examination showed absent perception of light on the right side with just perception of hand movements in the left eye along with corneal opacity; leukocoria in the right eye were also noted (Figure 1, ii-A). A general radiological survey for additional features by X-ray, echocardiogram and ultrasound of the chest showed evidence of dextrocardia with situs-inversus (Figure 1, ii-B). The X-rays based skeletal survey showed the presence of osteopenia with flaring of the metaphysial region.

Further radiological evaluation of cranial cavity using CT scan of the brain showed calcifications in bilateral thalamus, right parieto-occipital and left parietal regions (Figure 1, ii-C). Examination of the skull showed the presence of intracranial calcifications (Figure 1, ii-D). MRI (magnetic resonance imaging) of the brain revealed mixed intensity solid cystic lesion in the bilateral thalami (T1W image) (Figure 1, ii-E), T2 and flair hyperintensities involving bilateral frontal and parieto-occipital deep white matter, brainstem and bilateral middle cerebral peduncle. Several tiny cystic spaces were seen in the bilateral parieto-occipital regions. The right ocular globe showed evidence of retinal detachment (T2W) (Figure 1, ii-F-I). SWI phase image showed hypointense signal intensity in the areas of blooming consisted with the calcification changes (Figure 1, ii-J). Biochemical investigations: mildly deranged liver function indices with normal renal functions and electrolytes levels were observed. Complete blood cell counts showed mild leukopenia (total count = 3900 cells/cu mm) and thrombocytopenia (74,000 cells/cu mm). He has been receiving anti-spastic medications and physiotherapy for his motor illness. In summary, based on the presence of intracranial calcifications, symmetrical cystic lesions and clinical features of bilateral Coats disease of the eye (retinal telangiectasia and exudates) along with other extra-neurological manifestations, he was diagnosed with Coats plus syndrome.

## Methods

This research followed the tenets of the Declaration of Helsinki and the guidelines of the Indian Council of Medical Research, New Delhi. Following informed consent, peripheral blood DNA was extracted from the proband (case:II-1) and both the parents using a commercial kit, Wizard™ Genomic DNA Purification Kit(Promega, Madison, WI).

### Targeted resequencing of *CTC1*

The entire coding region and intron-exon junctions of the *CTC1* gene were amplified by polymerase chain reaction with appropriate primer pairs (Additional file 1: Table S1). The amplified PCR products were subjected to Sanger sequencing using ABIprism A370- sequencer (ABI, Foster City).

### Whole Exome Sequencing (WES)

Whole exome sequencing (WES) was carried out in three members of this family comprising the proband and both the parents. For exome sequencing, 2 μg of DNA was used for fragmentation, and DNA library preparation was carried out according to Illumina DNA sample prep protocol.v2. Exonic regions were captured and enriched using Truseq Illumina exome capture kit.v2 protocol. Cluster generation using cBOT was carried out for exome enriched libraries on Illumina flow cell.v3 followed by 100 bp paired end sequencing on Hiseq2000 using Illumina SBS kit.v3 protocol. Nearly 12 Gb raw data were generated for each sample. The base calls for all the sample reads were analyzed on CASAVA and subsequent pipeline for data processing is described in the following section. Whole exome sequencing analysis was performed as described before [9] with slight modifications at the level of raw read clipping (for details see Additional file 1: Table S2 and Text). From WES data, variation data (known and novel variations) for 15 candidate genes involved in telomere length maintenance activity (*TERF2IP, POT1, TERF1, OBFC1/STN1, CTC1, TERT, DKC1, WRAP53, TINF2, TPP1, TERF2, TEN1, TERC, NHP2* and *NOP10*) (Additional file 1: Table S3) and 28 genes associated with dextrocardia/situs-inversus phenotype were initially analyzed (Additional file 1: Tables S4 and S5). Following candidate gene analysis, the WES data was analyzed independently to obtain any likely disease associated candidate variation. The variant filtering protocol was performed essentially as described earlier [9].

In short, total variations (passing quality filters) → protein deleterious variations → Novel variants (absent in dbSNP/TGP) → homozygous/compound heterozygous variations → *In silico* predicted damaging variations.

### Homozygosity mapping

The combined variant calling file (vcf) of WES data of all the samples was analyzed for homozygosity mapping using the web based homozygosity mapper tool with default settings (http://www.homozygositymapper.org/).

### Southern blot based analysis of telomere length

9 μg of genomic DNA (for each sample) was subjected to overnight digestion by RsaI/HinfI (2U each). Digested DNA samples were run overnight at 50 V and transferred to a Nylon membrane, after denaturation and neutralization. The hybridization was done in Amersham Rapid Hyb buffer at 50°C, using the radio-labelled probe (CCCTAA)_4_. Post hybridization washes were done at 50°C, and the membrane was exposed to a Storage Phosphor Screen for overnight [10]. Imaging was done at Biorad PMI.

The validation of selected *CTC1* variant and 3′UTR variation of *HES7* was carried out by both Sanger sequencing using Big-Dye terminator sequencing kit and SNaPshot reaction kit (single base extension) for geotyping on 3130xl sequencer (ABI, Foster City) and was validated in DNA samples from 391 (for *CTC1*) and 370 (for *HES7*) healthy controls.

## Results and discussions

### Novel homozygous missense variation in *CTC1* observed through targeted resequencing

The proband described in this study has clinical manifestations which conform to the diagnostic criteria of Coats plus syndrome such as bilateral exudative retinopathy, intracranial calcification and cystic lesions along with non-neurological features like premature graying of hair, café-au-lait spots, osteopenia and metaphysical flaring. Neurologically, the patient only had spasticity and left hemiparesis with no history of seizures, ataxia or dystonia (the later features have been reported in previously described cases of Coats plus). The patient showed an unusual feature of abnormal right-left patterning of the internal organs demonstrated as dextrocardia with situs inversus which has not previously been described as a manifestation of Coats plus clinically. Based on prominent clinical features of Coats plus in our patient (II-1), we carried out a targeted search for *CTC1* mutations by sequencing of the exonic regions. This led us to identify a novel homozygous DNA variation, c.1451A > C (p.H484P) in exon 9 of the *CTC1* gene (Figure 1, iii). As expected, both his parents (I-1 and I-2) were heterozygous for this change (Figure 1, iii). Although the parents were nonconsanguineously married, Indian large ethnic population groups are known to have endogamous marriage patterns which may explain the observed homozygosity of the *CTC1* variation in this patient. To best of our knowledge, this variation has not been reported as a polymorphic locus or associated with disease in the current literature. A synonymous variation has been described at the same amino acid position (chr17:8138232 A > G; p.H484H) in the recently available dataset from Exome Aggregation Consortium (ExAC), Cambridge, MA (http://exac.broadinstitute.org). (It is one of the largest databases and catalogues variations from 61,486 exome data). In the literature to date, all the reported *CTC1* mutations either with Coats plus syndrome or with DC phenotype have been observed as compound heterozygous changes comprising at least one protein truncating mutation (frameshift or nonsense). In order to gather further evidence to substantiate the sole pathogenicity of the observed homozygous p.H484P change in *CTC1* in our patient and to understand the complex association of situs inversus, we extended our search to identify other genetic defects through WES.

### Whole exome sequencing analysis showed additional rare variations and a 3 Mb homozygous stretch containing *CTC1* (p.H484P) and a rare 3′UTR *HES7* variation

Initial analysis of the WES data included a targeted search for rare variations in candidate genes involved in telomere length maintenance. This analysis was conducted to rule out the involvement of any other genes related to telomere biology in the pathogenesis of Coats plus. We observed 23 variations (novel and known) in 9 genes in the patient and his parents, but none segregated with Coats plus using both homozygous and compound heterozygous recessive model except for the *CTC1* p.H484P variant identified using sanger sequencing (Additional file 1: Table S3).

Next, the variation analysis of 28 dextrocardia/situs-inversus candidate genes identified 9 known 3′UTR variations in accordance with homozygous recessive disease model in HES7, GATA4 and SMAD2 genes. None of the variants identified fitted the compound heterozygous model (Additional file 1: Table S5). Among all the identified homozygous variations, a 3′UTR variation rs182882481 (c.*556 T > C, HES7) appeared to be relevant to the dextrocardia phenotype due to the fact that mutations in HES7 (Hairy and Enhancer of Split 7) have previously been described as a cause of Spondylocostal Dysostosis 4 (SCDO4) with dextrocardia. Despite the fact that rs182882481 is present on the dbSNP database, we propose that this variation may be pathogenic since, the reported minor allele frequency is 0.092% (2/2179) and there are no homozygous genotypes reported on the TGP (1000Genome project) database.

Following this targeted search, we analyzed all the exonic variations obtained from our WES data in an unbiased manner to identify potential deleterious variations that are likely to explain the complex Coats plus syndrome phenotype with dextrocardia/situs inversus. The variant filtering (as described above in the methodology section) finally yielded us the same p.H484P change in *CTC1* along with three other likely damaging variations in *TEK, PER1,* and *MUC17* (Table 1). The CHet analysis showed variations in three genes (*DNHD1*, *SIVA1* and *KMT2C*) segregating with the disease status (Additional file 1: Table S6). Based on the literature and functional relevance of other variations identified from our WES data, only p.H484P change in the *CTC1* gene was suggestive of likely candidate status for Coats plus syndrome phenotype in this patient.

**Table 1:** **Whole exome analysis of variation data with autosomal recessive homozygous model in this family with coats plus syndrome and dextrocardia phenotype**

	**II-1(proband)**	**I-1(Father)**	**I-2(Mother)**
Number of variations called(Pass/total)	70060/78946		
number of variations per subject	54353	53466	51118
Novel variations(not reported in dbSNP135,TGP) count Total(homo/het)	1696(128/1568)	1700(143/1557)	1530(115/1415)
Novel protein deleterious variations(homo/het)	370(20/350)	381(19/362)	360(345/15)
Autosomal recessive homozygous model filtered variations	7		
SNV	5(*CTC1,NKTR,PER1,TEK,ZBTB4*)		
ins/del	2(*FAM157A,MUC17*)		
Deleterious variations after computational prediction	4		
SNV	*CTC1*(p.H484P),*PER1*(p.A772T),*TEK*(p.E103D)		
ins/del	*MUC17*(Q1903Hfs*13)		
Putative variation explaining Coats plus phenotype	**c.1451A > C; p.H484P(** ***CTC1*** **)**		

SNV; single nucleotide variations.

Bold indicates candidate variation prioritized (after applying filtering strategy) after WES analysis associated with Coats plus syndrome.

We anticipated a region of extended homozygosity around p.H484P (*CTC1*) which could contain some additional pathogenic variation. Homozygosity mapping analysis identified a large homozygous stretch (~3 Mb) on chr17 containing both *CTC1* p.H484P and HES7 rs182882481 (Additional file 1: Table S7). This finding provides a possible explanation for the unusual association of Coats plus with dextrocardia.

The p.H484P (*CTC1*) change was absent in 740 chromosomes and *HES7* variation (rs182882481) showed a frequency of 0.1% (1:782 chromosomes) from ethnicity matched controls where one control subject harbored a heterozygous genotype.

### Plausible pathogenicity of p.H484P (*CTC1*) with Coats plus syndrome

**Figure 2:**
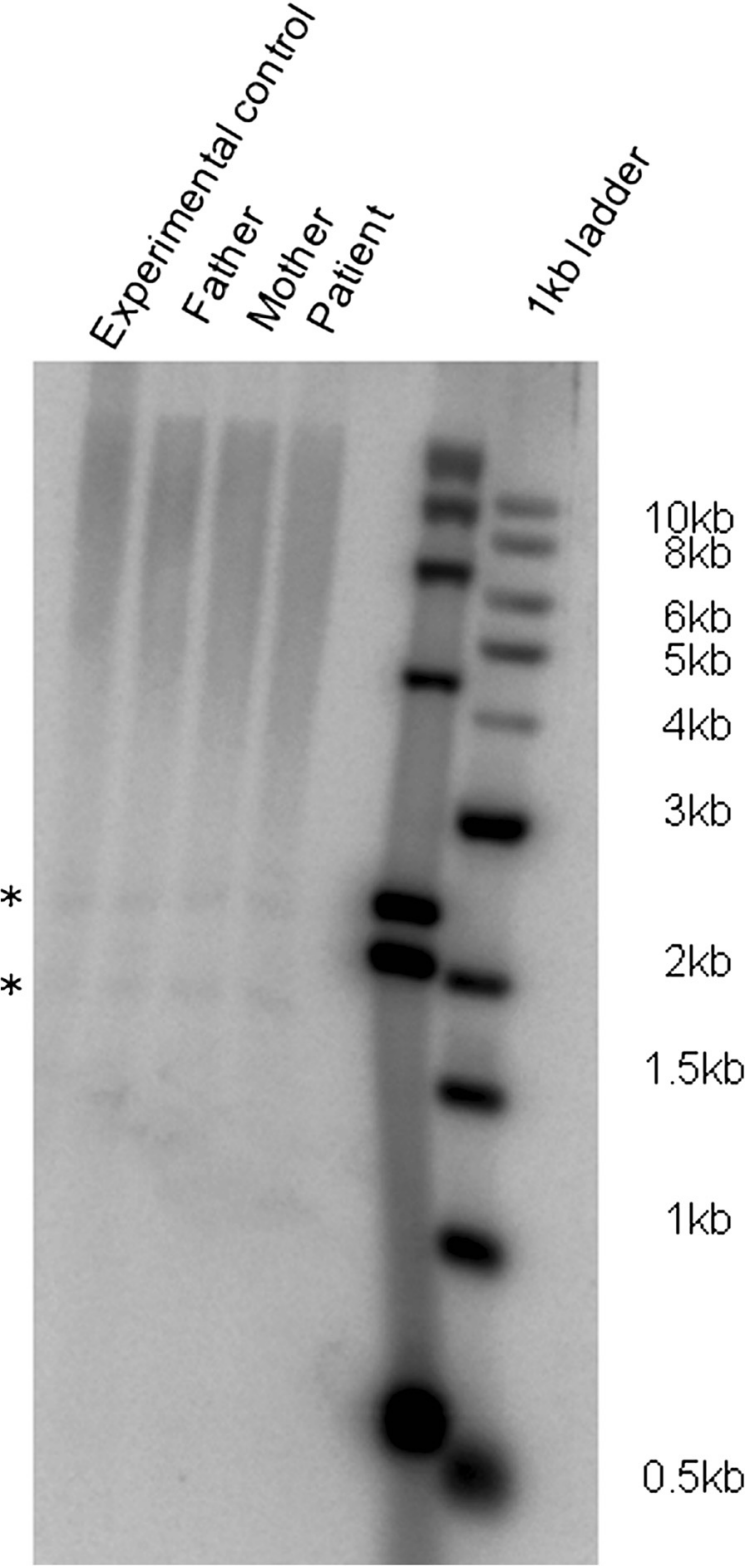
**Southern blot analysis to show the shortening of telomere length in the affected individual.** The telomere length in father and mother is 10 Kb, whereas it is 8 Kb in the affected individual. The telomere length was measured by the Bio-rad Software One Density Plot. The asterisk shows internal TTAGGG repeats, as a loading control.

To elucidate the functional impact of the identified missense *CTC1* variation, we carried out Southern blot analysis. The patient’s DNA showed a reduction in the telomere length compared to that of his parents (Figure 2).

Based on the following five criteria, we considered p.H484P as a likely disease associated variation responsible for Coats plus syndrome phenotype and for genetic diagnosis: 1) This variation segregated with the disease following a recessive mode of inheritance. 2) Telomere length is significantly reduced in the affected individual. 3) Histidine residue is conserved across different *CTC1* orthologs (Figure 1, iv). 4) Insilico analysis predicted this mutation/change to be of damaging nature (Polyphen-2, score of 0.998). 5) It is absent in the 370 ethnicity matched control subjects that we have genotyped, dbSNP, 1000 Genome, Exome Variant Server and ExAC database.

The causative gene *CTC1* (conserved telomere maintenance component 1 [#MIM 613129] for Coats plus syndrome was identified independently by Anderson et al. and Polvi et al. [2,4]. *CTC1*, located on chromosome 17p13.1, contains 23 exons and codes for a 1,217 amino acid long protein (http://www.genome.ucsc.edu). This protein interacts with STN1 (OBFC1/oligonucleotide/oligosaccharide-binding fold containing 1 [#MIM 613128]) and TEN1 (telomerase capping complex subunit homolog (*S. cerevisiae*) [#MIM 613130]) to form a trimeric CST complex [11]. The CST complex, but not its individual components, binds to single-stranded(ss) DNA with high affinity in a sequence independent manner. The complex associates constantly with a fraction of telomeres, suggesting that it might be protecting telomeres from lethal degradation [11]. *CTC1* has two oligonucleotide/oligosaccharide-binding(OB) folds in its N-terminal half and a third OB fold near the C terminus; the former mainly contributes to the DNA binding activity of *CTC1* as OB folds are typically found in proteins that bind to ssDNA, whereas the C-terminal OB fold is responsible for the CST complex formation through interaction with STN1 [11]. The p.H484P mutation reported in our patient is located in the N-terminal 700 amino acid region which is important for the binding of *CTC1* to ssDNA through its two OB domains [11]. We speculated that the p.H484P mutation might induce conformation change in the *CTC1* protein, thus reducing its binding to ssDNA and in turn, compromising the structural integrity of the telomeres. This was further corroborated by the shortened telomere length in the affected subject (in line with the previous reports of shortened telomeres in individuals with *CTC1* mutations).

### Molecular links of dextrocardia/situs-inversus phenotype in Notch signaling target genes

We observed a novel homozygous genotype in the 3′UTR of *HES7*. Missense mutations of *HES7* have been reported to cause Spondylocostal Dysostosis 4 (SCDO4) [12]. A duplication mutation (c.400_409dupAAACCGCCCC) in *HES7* has been reported to cause dextrocardia with situs inversus in three out of four individuals showing SCOD4 manifestations, suggesting an incomplete penetrance of this left-right patterning defect [13]. In our patient, we did not find any skeletal deformities (abnormal vertebral segmentation, ribs fusions, shortened trunk etc.) that are usually reported with SCOD4. The null *HES7*-mice show disruption of anterior-posterior polarity but no disruption of left-right patterning [13]. Unfortunately, we cannot show direct functional consequence of rs182882481 homozygous variation on *HES7*, but we speculate the role of this variation on *HES7* mRNA stability and its effect on subsequent downstream signaling pathway. It is known that *HES7* is a transcriptional repressor of *LFNG* (Lunatic Fringe) and an autorepressor. *LFNG* also regulates Notch signaling through a feedback loop [14]. Their cyclic gene expression plays an important role in the segmentation of the presomitic mesoderm (PSM) and 3′UTRs of both the genes are critical determinants of cyclical pattern of their expression by controlling mRNA stability [14]. Both the genes are activated by Notch signaling and Notch signaling plays an important role in the left-right pattern determination during embryogenesis and periodic somatic segmentation [15,16].

We also observed a missense homozygous variation (p.E103D) in *TEK* gene (Tyrosine kinase, endothelial) (Table 1) which is also a target of Notch signaling. However, we found the same *TEK* variation in a heterozygous state in one healthy control subject from our in-house generated whole exome data set (35 ethnicity matched controls) and it has also been reported as a very rare variation (0.08948%) in ExAC database. *TEK*, a receptor protein with its ligand *ANGPT1*, promotes vasculogenesis through Notch signaling [17]. Missense mutations in *TEK* have been reported to cause cutaneomucosal venous malformation (VMCM), which follow an autosomal dominant mode of inheritance. Some patients with *TEK* mutation manifest ventricular septal defect, suggesting its role in cardiac development. However, direct role of *TEK* has not been implicated with either Coats plus syndrome or dextrocardia phenotype till date. Notch signaling regulates cardiac development, septal development and coronary vascular development [18]. Cardiac septal abnormalities, like atrial septal defect, ventricular septal defect and coronary artery fistula have been reported in a few cases of Coats plus syndrome harboring compound heterozygous mutations in *CTC1* [4]. This may also be suggestive of the role of *CTC1* in Notch signaling. Therefore, it is possible that the overall complex association of Coats plus syndrome and dextrocardia/situs-inversus might be the resultof multiple genetic factors comprising *CTC1*, *HES7* and *TEK* and their cross talk through Notch signaling deregulation. However, it is very well possible that the p.H484P change of *CTC1* may be the sole determinant of the pathophenotype in our patient.

## Conclusions

This is the first study to report a genetically confirmed case of Coats plus syndrome from India. It also reports the association of a homozygous *CTC1* missense mutation p.H484P with Coats plus syndrome. The genetic analysis using WES in this family facilitated the identification as well as establishment of the putative role of genetic variations for such a complex phenotype. The present finding will be useful for genetic diagnosis and carrier detection in the family and for other patients with similar disease manifestations.

### Ethics statement

This research followed the tenets of the Declaration of Helsinki and the guidelines of the Indian Council of Medical Research, New Delhi. A written informed consent was obtained from the parents for publication of this research article and any accompanying images. A copy of the written consent is available for review by the Editor of this journal.

### Data availability

All the supporting information/raw data will be available freely to any researcher without breeching the confidentiality of the family for the non commercial usage.

## Additional file

Additional file 1: Table S1. Details of PCR primers used in the mutation analysis of *CTC1.* **Table S2.** Details of Exome sequencing with respect to read counts, depth of coverage and mapping statistics for each DNA sample sequenced. **Table S3.** Variation analysis of known telomere associated genes. **Table S4.** WES variations from known Dextrocardia/situs-inversus associated genes. **Table S5.** Variation analysis of known candidate of dextrocardia phenotype with homozygous disease model. **Table S6.** Compound heterozygous model WES data. **Table S7.** The variation found in the homozygous stretch on chr17.
