# Supplementary material for: Whole exome sequencing in an Indian family links Coats plus syndrome and dextrocardia with a homozygous novel CTC1 and a rare HES7 variation
Source: BMC Med Genet. 2015 Feb 10;16:5. doi: 10.1186/s12881-015-0151-8 (PMC4422476; doi:10.1186/s12881-015-0151-8)
Supplement: Additional file 1: Table S1. — Details of PCR primers used in the mutation analysis of CTC1. Table S2. Details of Exome sequencing with respect to read counts, depth of coverage and mapping statistics for each DNA sample sequenced. Table S3. Variation analysis of known telomere associated genes. Table S4. WES variations from known Dextrocardia/situs-inversus associated genes. Table S5. Variation analysis of known candidate of dextrocardia phenotype with homozygous disease model. Table S6. Compound heterozygous model WES data. Table S7. The variation found in the homozygous stretch on chr17. [file 12881_2015_151_MOESM1_ESM.docx]

**Supplementary Data**

**Whole exome sequencing in an Indian family links Coats plus syndrome and dextrocardia with a homozygous novel *CTC1* and a rare *HES7* variation**

Manjunath Netravathi^1^, Renu Kumari^2$^, Saketh Kapoor^3$^, Pushkar Dakle^2^, Manish Kumar Dwivedi^2^, Sumitabho Deb Roy^4^, Paritosh Pandey^5^, Jitender Saini^6^, Anil Ramakrishna^1^ , Devaraddi Navalli^1^, Parthasarathy Satishchandra^1^, Pramod Kumar Pal^1*^ , Arun Kumar^3*^ and Mohammed Faruq^2*^

^1^Department of Neurology, National Institute of Mental Health and Neuro Sciences, Bangalore, India.

^2^Genomics and Molecular Medicine, CSIR-Institute of Genomics and Integrative Biology, Mall Road, New Delhi, India.

^3^Department of Molecular Reproduction, Development and Genetics, Indian Institute of Science, Bangalore, India.

^4^Proteomics and Structural Biology Unit, CSIR-Institute of Genomics and Integrative Biology, Mall Road, New Delhi, India.

^5^Department of Neurosurgery, National Institute of Mental Health and Neuro Sciences, Bangalore, India.

^6^Department of Neuroimaging and Interventional Radiology, National Institute of Mental Health and Neuro Sciences, Bangalore, India.

**Supplementary materials and methods**

**Genetic analysis of the family**

Following informed consent, 3-5 ml of the peripheral blood sample was collected from proband(case:II-1) and both the parents in a Vacutainer EDTA™ tube (Beckton-Dickinson, Franklin Lakes, NJ) for genomic DNA isolation using a Wizard™ Genomic DNA Purification Kit (Promega, Madison, WI). This research followed the tenets of the Declaration of Helsinki and the guidelines of the Indian Council of Medical Research, New Delhi.

**Targeted resequencing of *CTC1***

For mutation screening, the entire coding region and intron-exon junctions of the *CTC1* gene (GenBank NM_025099.5) were amplified by polymerase chain reaction with appropriate primer pairs (Table S1). The amplified PCR products were subjected to Sanger sequencing followed by analysis on an ABIprism A370-automated sequencer (PE Biosystems, Foster City, CA). Once the mutation was identified, both parents were also examined for the presence of the mutation by sequencing. A total of 50 normal controls were examined for the presence of the mutation by DNA sequencing.

**Whole exome sequencing**

Whole exome sequencing was carried out in 3 individuals of this family comprising the proband and both the parents. For exome sequencing, 2 μg of DNA was used for fragmentation, and DNA library preparation was carried out according to Illumina DNA sample prep protocol.v2. Exonic regions were captured and enriched using Truseq Illumina exome capture kit.v2 protocol. Cluster generation using cBOT was carried out for exome enriched libraries on Illumina flow cell.v3 followed by 100bp paired end sequencing on Hiseq2000 using Illumina SBS kit.v3 protocol. Nearly 12 Gb raw data was generated for each sample. The base calls for all the sample reads were analyzed on CASAVA and subsequent pipeline for data processing is described in the following section. The validation of selected candidate variation for dextrocardia (HES7, rs182882481) from exome analysis was carried out by Sanger sequencing using Big-Dye terminator sequencing kit on 3130xl sequencer (ABI, Foster City).

**Exome sequence analysis**

Paired-end raw sequencing reads of length 101 bp were trimmed to 96 bp using Trimmomatic^1^ based on the quality score assessment by FastQC [<http://www.bioinformatics.babraham.ac.uk/projects/fastqc/>]. These reads were further trimmed if the phred quality score dropped below 20 for a window of 25 bases. For subject, I-2(pedigree chart, Fig. 1, i), the raw reads were trimmed with a more stringent cutoff of phred score dropping below 30 in a window of 10 bases. Finally, the reads which were of length greater than 50bp were taken forward. The filtered reads were then aligned against human genome reference (human.g1k.v37.fasta) using Stampy^2^. PCR duplicates were labeled in aligned reads using Picard Markduplicates ([http://picard.sourceforge.net/)](http://picard.sourceforge.net/).

Genome Analysis toolkit (GATK) from http://www.broadinstitute.org/gatk was used to realign BAM file around indels and further scores were re-calibrated to obtain final processed BAM file. Variant calls were made jointly for all the samples using Unified Genotyper module of GATK^3^. Raw calls were labeled using GATK's VariantAnnotator based on filter expressions. SeattleSeqAnnotation138 server was used to further annotate the calls based on their genomic location– genic/non-genic and their effect(<http://snp.gs.washington.edu/SeattleSeqAnnotation138/>).

**Analysis of variations in the known candidate genes from WES data**

The variation data was obtained for 15 genes involved in telomere length maintenance activity(*TERF2IP,POT1,TERF1,OBFC1/STN1,CTC1,TERT,DKC1,WRAP53,TINF2,TPP1,TERF2,TEN1,TERC,NHP2* and *NOP10*). All the known and novel variations passing quality filter criterias of GATK were considered for the further analysis(n=23 variations) of direct or indirect association. For Dextrocardia phenotype, a total of 111 variations were obtained from 28 known candidate genes; *ACVR2B, CCDC103, CRELD1, DNAH11, GATA4, HES7, MEGF8, NODAL, PKD2, PRKAR1A, SMAD2, CCDC11, INVS, NPHP3, DNAI1, DNAAF1, CCDC114, DYX1C1, CLMP, UBR1, SCN5A, B9D1, CFC1, DNAAF3, ZIC3, MBS1, GDF1, ZMYND10* (**supplementary TableS4**). All dextrocardia genes related variations were also analyzed with autosomal recessive inheritance pattern to document their potential association with the phenotype (**supplementary Table S5**).

**Whole exome variants analysis and variant filtering**

For a stringent and unbiased approach to analyze the whole exome variations in this family, we filtered variations in the following manner. All calls that were reported in dbsnp138 (<http://www.ncbi.nlm.nih.gov/projects/SNP/index.html>) were removed. Only protein damaging variations, such as non-synonymous, stop-gain or loss, splice site and indels, were further used to locate variations/gene that fit the model of recessive inheritance i.e genes with homozygous or compound heterozygous mutations in same gene.

Finally, these calls were predicted for their role in pathogenicity using SIFT (<http://sift.jcvi.org>), Polyphen-2 algorithm (<http://genetics.bwh.harvard.edu/pph2/>) and Mutation Taster ([www.mutationtaster.org/](http://www.mutationtaster.org/)). To find the functional significance of the mutated amino acid residue, CTC1 protein sequences from different species were aligned by the ClustalW2 program (<http://www.ebi.ac.uk/Tools/msa/clustalw2/>).

To predict the effect of the mutation on CTC1 function, we used two bioinformatics programs: Mutation Taster (http://www.mutationtaster.org) and PolyPhen-2 (http://genetics.bwh.harvard.edu/pph2/). The output from the Mutation Taster program is a p (probability) value. A p value close to 1 indicates a high 'security' of prediction function. The output score from the PolyPhen-2 program ranges from 0 to a positive number, where 0 is neutral, and a high positive number is damaging to protein function.

**Supplementary Table S1:** Details of PCR primers used in the mutation analysis of *CTC1*.

| Exon | Primer Sequence (5’ to 3’) | T_m_ (°C) | Amplicon size (bp) |
| --- | --- | --- | --- |
| 1 | F:CCTCGCGGAGTCTCTAGGAAGCGAG | 62 | 321 |
|  | R:ACAACCCCCTCACCCATGGGCCGGA |  |  |
| 2 | F:CCGGCCCGGATATTTTTTTCTAGTC | 62 | 381 |
|  | R:AGACTACCTTGTCCTCCGCTCAC |  |  |
| 3 | F:ATGTTCCCTGCAACCTTTCCCTG | 62 | 443 |
|  | R:ACCTCCCCGTTTGTTTCCTCAG |  |  |
| 4 | F:CAAGAATGGTGTCCAAGGATCAAGGCA | 62 | 460 |
|  | R:TCAAAGCCCACCATTCCCTTGATTTGGA |  |  |
| 5 | F:TGAGACGTAGTTTCACTCCTGCTGC | 62 | 384 |
|  | R:TCCTACATGGCTCCTCCTATGATGC |  |  |
| 6+7 | F:GTCTCTGAGTTCAAATCGTGAGG | 62 | 700 |
|  | R:ATTTCTATATAACCACCCTGCTGC |  |  |
| 8 | F:GGCAGTGGCAAAGTGCGGTTAACTC | 62 | 410 |
|  | R:GAGCCTGCCTCTTCAAGAATCCTGC |  |  |
| 9+10 | F:CAGTGGGAGTAGCACACTTCCTTGG | 62 | 659 |
|  | R:ATGACGAAGAAAGATAGTAACCGAGAC |  |  |
| 11 | F:AGCTTTACCATGTAATACAGCATCTGG | 62 | 322 |
|  | R:TCTGCATCTCTAGTAAATCCCAGCAG |  |  |
| 12 | F:GTGAGGCCGGTAGGATGGTTTGC | 62 | 346 |
|  | R:ACTGGACATAGACTCTGTTGGGAG |  |  |
| 13+14 | F:TCCTTGGGGTCATGGTTGTCTTGAG | 62 | 724 |
|  | R:AGGCCTAGAGAATGACCAGGCACTG |  |  |
| 15 | F:CAGTGCCTGGTCATTCTCTAGGCCTC | 62 | 453 |
|  | R:CTTCACCAAACCCAGCTAGCTTGGGA |  |  |
| 16+17 | F:TGAAGTTCTAGCAGATACTTTAGCTCCA | 59 | 567 |
|  | R:TATGAAAGAGAAGGGGTCGCTGCAG |  |  |
| 18 | F:CCATGGCTAGGATATGTCCCTCAG | 62 | 306 |
|  | R:GGATTGGAGAAGACTGTAATGATGG |  |  |
| 19+20 | F:CTCTAAGTCCTTCTAGACCCAC | 62 | 495 |
|  | R:TTCTCATGAGCCAGGAATTATGC |  |  |
| 21+22 | F:GTGAGCCAAAGACTGCAGACAGC | 62 | 595 |
|  | R:GAAATTCCCCATCAGTCCCTCATC |  |  |
| 23 | F:GATGAGGGACTGATGGGGAATTTC | 62 | 349 |
|  | R:CTGGAGTCCTTGGTTCAATCACAGA |  |  |
| Genotyping primer* for p.H484P variation | F: GTAGGAGCCAGGAGTTGCA  R: TTGGCAAGGAGAGCAGGATT  IP: GCTCCCAGGAGAGGAATGTT | 60 | 164 |

- *Genotyping was done by SNaPshot reaction kit (ABI) which is based on single base extension chemistry. F, forward primer; R, reverse primer; IP, Internal primer targeted penultimate to the variation of interest.

**Supplementary Table S2:** Details of Exome sequencing with respect to read counts, depth of coverage and mapping statistics for each DNA sample sequenced.

| Sample ID | Raw  Reads | Filtered Reads | Alignment  (Properly Paired) | mean depth | Percentage of Exome with non zero coverage  (per base) |
| --- | --- | --- | --- | --- | --- |
| II-1(Proband) | 71486240 | 66121038 | 99.64% | 59.56 | 95.05 |
| I-1(Father) | 63329178 | 57945272 | 99.21% | 50.60 | 94.93 |
| I-2(Mother) | 67592800 | 29144024 | 99.50% | 22.12 | 93.48 |

**Supplementary Table S3:** Variation analysis of known telomere associated genes.

| Genomic coordinates | Variation status | GL | FG | II-1 | I-1 | I-2 | ExAC allele frequency |
| --- | --- | --- | --- | --- | --- | --- | --- |
| **chr17:g.8138233T>G** | **Novel** | ***CTC1*** | **missense** | **1/1** | **0/1** | **0/1** | #N/A |
| chr17:g.8129160A>G | rs8078338 | *CTC1* | 3'UTR | 0/0 | 0/0 | 0/1 | #N/A |
| chr17:g.8132763T>C | rs3826543 | *CTC1* | missense | 0/0 | 0/0 | 0/1 | 0.7511 |
| chr17:g.8135061T>C | rs3027238 | *CTC1* | missense | 1/1 | 1/1 | 1/1 | 0.9508 |
| chr17:g.7606723GC>G | rs373064567 | *WRAP53* | frameshift | 0/0 | 0/0 | 0/1 | 0.001833 |
| chr15:g.34634033A>G | rs3063 | *NOP10* | 3'UTR | 0/1 | 0/1 | 0/0 | #N/A |
| chr15:g.34634124C>G | rs1045238 | *NOP10* | 3'UTR | 0/1 | 0/1 | 0/0 | 0.1781 |
| chr15:g.34634138G>A | rs1045204 | *NOP10* | 3'UTR | 0/1 | 0/1 | 0/0 | 0.1666 |
| chr15:g.34634139T>C | rs1045194 | *NOP10* | 3'UTR | 0/1 | 0/1 | 0/0 | 0.1783 |
| chr10:g.105638232T>C | rs4917405 | *OBFC1* | 3'UTR | 1/1 | 1/1 | 1/1 | #N/A |
| chr10:g.105640978C>T | rs7100920 | *OBFC1* | 3'UTR | 0/1 | 0/1 | 1/1 | #N/A |
| chr10:g.105641935G>GCAAGTGA | rs147944736 | *OBFC1* | 3'UTR | 0/1 | 0/1 | 1/1 | #N/A |
| chr10:g.105657316G>C | rs10786775 | *OBFC1* | missense | 1/1 | 1/1 | 1/1 | 0.9068 |
| chr10:g.105659826T>C | rs2487999 | *OBFC1* | missense | 1/1 | 1/1 | 1/1 | 0.9069 |
| chr7:g.124462655T>C | rs76436625 | *POT1* | 3'UTR | 0/0 | 0/0 | 0/1 | 0.189 |
| chr7:g.124462661C>T | rs17246404 | *POT1* | 3'UTR | 0/1 | 0/0 | 0/1 | #N/A |
| chr8:g.73958718G>A | rs10112752 | *TERF1* | 3'UTR | 0/0 | 0/0 | 0/1 | #N/A |
| chr16:g.75681743C>G | rs1865493 | *TERF2IP* | 5'UTR | 0/1 | 0/1 | 0/1 | 0.2544 |
| chr16:g.75691252A>G | Novel | *TERF2IP* | 3'UTR | 0/0 | 0/1 | 0/0 | #N/A |
| chr5:g.1268721C>T | Novel | *TERT* | synonymous | 0/1 | 0/1 | 0/0 | 0.00009147 |
| chr5:g.1294086C>T | rs2736098 | *TERT* | synonymous | 0/1 | 1/1 | 0/0 | 0.4008 |
| chrX:g.153994596G>T | rs2728532 | *DKC1* | synonymous | 1/- | 1/- | 1/1 | 0.9978 |
| chrX:g.154005148G>A | rs1800533 | *DKC1* | 3'UTR | 1/- | 0/- | 0/1 | 0.1811 |

**Supplementary Table S4: WES variations from known Dextrocardia/situs-inversus associated genes.**

| **Coordinates** | **dbSNP** | **Gene** | **II-1** | **I-1** | **I-2** | ExAC allele frequency |
| --- | --- | --- | --- | --- | --- | --- |
| chr3:38519424 A>G | rs2070489 | *ACVR2B* | 1/1 | 1/1 | 1/1 | 0.5681 |
| chr3:38524742 C>T | rs1046048 | *ACVR2B* | 1/1 | 1/1 | 1/1 | 0.485 |
| chr3:38525567 T>C | rs7642472 | *ACVR2B* | 1/1 | 1/1 | 1/1 | #N/A |
| chr3:38525734 C>T | rs6599204 | *ACVR2B* | 1/1 | 1/1 | 1/1 | #N/A |
| chr3:38525864 A>C | rs7640050 | *ACVR2B* | 1/1 | 1/1 | 1/1 | #N/A |
| chr3:38526166 CAA>C | rs56291663 | *ACVR2B* | 1/1 | 1/1 | 1/1 | #N/A |
| chr3:38527215 T>C | rs11914389 | *ACVR2B* | 1/1 | 1/1 | 1/1 | #N/A |
| chr3:38527913 C>T | rs11926767 | *ACVR2B* | 1/1 | 1/1 | 1/1 | #N/A |
| chr3:38528537 G>T | rs928813 | *ACVR2B* | 1/1 | 1/1 | 1/1 | #N/A |
| chr3:38529440 C>G | rs6599205 | *ACVR2B* | 1/1 | 1/1 | 1/1 | #N/A |
| chr3:38529818 C>T | rs7433277 | *ACVR2B* | 1/1 | 1/1 | 1/1 | #N/A |
| chr3:38529825 C>T | rs2370840 | *ACVR2B* | 1/1 | 1/1 | 1/1 | #N/A |
| chr3:38531211 T>G | rs7374458 | *ACVR2B* | 1/1 | 1/1 | 1/1 | #N/A |
| chr3:38532511 G>A | rs1058945 | *ACVR2B* | 1/1 | 1/1 | 1/1 | #N/A |
| chr3:38533335 A>C | rs13072731 | *ACVR2B* | 1/1 | 1/1 | 1/1 | #N/A |
| chr3:38534133 T>TA | rs34717882 | *ACVR2B* | 1/1 | 1/1 | 1/1 | #N/A |
| chr17:42980189 G>A | rs8079308 | *CCDC103* | 0/1 | 0/0 | 1/1 | 0.3266 |
| chr18:47777323 T>C | Novel | *CCDC11* | 0/0 | 0/1 | 0/0 | 0.00001635 |
| chr19:48799716 A>G | rs2292112 | *CCDC114* | 0/1 | 0/0 | 0/1 | #N/A |
| chr11:122943495 T>C | rs7949414 | *CLMP* | 0/0 | 0/1 | 0/0 | #N/A |
| chr11:122944081 C>CCT | rs149599709 | *CLMP* | 0/0 | 0/1 | 0/0 | #N/A |
| chr11:123065902 A>G | rs3132824 | *CLMP* | 1/1 | 1/1 | 1/1 | #N/A |
| chr3:9976159 A>G | rs279552 | *CRELD1* | 1/1 | 1/1 | 1/1 | 0.9916 |
| chr3:9987049 A>G | rs73118375 | *CRELD1* | 1/1 | 1/1 | -/- | #N/A |
| chr16:84203612 A>G | rs17856705 | *DNAAF1* | 0/1 | 0/1 | 0/1 | 0.4 |
| chr16:84203730 G>C | rs9972733 | *DNAAF1* | 0/0 | 0/1 | 0/0 | 0.09698 |
| chr16:84203939 C>T | rs11644164 | *DNAAF1* | 0/1 | 0/1 | 0/1 | 0.3424 |
| chr16:84209634 C>G | rs2288019 | *DNAAF1* | 0/1 | 0/1 | 0/1 | 0.5276 |
| chr16:84209738 T>C | rs2288020 | *DNAAF1* | 0/1 | 0/1 | 0/1 | 0.3377 |
| chr16:84209816 T>C | rs2288022 | *DNAAF1* | 0/1 | 0/1 | 0/1 | 0.3365 |
| chr16:84209864 G>C | rs2288023 | *DNAAF1* | 0/1 | 0/1 | 0/1 | 0.3379 |
| chr7:21582963 G>T | rs2285943 | *DNAH11* | 0/0 | 0/0 | 0/1 | 0 |
| chr7:21582964 A>T | rs2285944 | *DNAH11* | 0/0 | 0/0 | 0/1 | 0 |
| chr7:21598500 A>G | rs72655972 | *DNAH11* | 0/0 | 0/0 | 0/1 | 0 |
| chr7:21599233 C>T | rs10950854 | *DNAH11* | 0/1 | 1/1 | 0/1 | 0 |
| chr7:21603886 A>G | rs4392792 | *DNAH11* | 0/1 | 0/1 | 0/0 | 0 |
| chr7:21628197 A>G | rs12670130 | *DNAH11* | 0/0 | 0/1 | 0/0 | 0 |
| chr7:21628237 C>T | rs6963535 | *DNAH11* | 1/1 | 1/1 | 1/1 | 0 |
| chr7:21628242 C>G | rs62441683 | *DNAH11* | 0/0 | 0/1 | 0/0 | 0 |
| chr7:21630982 A>G | rs4615458 | *DNAH11* | 0/0 | 0/1 | 0/1 | 0 |
| chr7:21640361 T>C | rs10269582 | *DNAH11* | 0/1 | 0/0 | 0/1 | 0 |
| chr7:21640405 A>G | rs10224537 | *DNAH11* | 0/1 | 0/1 | 1/1 | 0 |
| chr7:21641218 G>A | rs3827657 | *DNAH11* | 0/1 | 0/1 | 1/1 | 0 |
| chr7:21659645 T>C | rs56029521 | *DNAH11* | 0/1 | 0/1 | 0/1 | 0 |
| chr7:21765452 C>T | rs12536928 | *DNAH11* | 1/1 | 1/1 | 0/1 | 0 |
| chr7:21775443 G>A | rs2072221 | *DNAH11* | 1/1 | 0/1 | 1/1 | 0 |
| chr7:21778449 C>T | rs1109806 | *DNAH11* | 0/1 | 0/0 | 1/1 | 0 |
| chr7:21779278 A>G | rs9639393 | *DNAH11* | 0/1 | 0/0 | 1/1 | 0 |
| chr7:21824058 A>G | Novel | *DNAH11* | 0/0 | 0/1 | 0/0 | 0 |
| chr7:21893993 G>T | rs4722064 | *DNAH11* | 0/1 | 0/0 | 0/1 | 0 |
| chr7:21901566 T>C | rs4722067 | *DNAH11* | 0/1 | 0/1 | 1/1 | 0 |
| chr7:21924014 A>G | rs6461613 | *DNAH11* | 1/1 | 1/1 | 1/1 | 0 |
| chr7:21932044 C>T | rs12537531 | *DNAH11* | 1/1 | 1/1 | 1/1 | 0 |
| chr9:34500821 G>A | rs11793196 | *DNAI1* | 0/1 | 0/0 | 0/1 | 0.1564 |
| chr8:11614575 A>G | rs3729856 | *GATA4* | 0/1 | 0/0 | 0/1 | 0.09638 |
| chr8:11616338 A>C | rs867858 | *GATA4* | 1/1 | 0/1 | 0/1 | #N/A |
| chr8:11616410 C>T | rs1062219 | *GATA4* | 0/0 | 0/1 | 0/1 | #N/A |
| chr8:11616501 T>C | rs884662 | *GATA4* | 0/0 | 0/1 | 0/1 | #N/A |
| chr8:11616516 T>C | rs904018 | *GATA4* | 1/1 | 1/1 | 1/1 | #N/A |
| chr8:11616547 C>G | rs12825 | *GATA4* | 1/1 | 0/1 | 0/1 | #N/A |
| chr8:11616571 A>G | rs804291 | *GATA4* | 1/1 | 1/1 | 1/1 | #N/A |
| chr8:11616836 G>A | rs804290 | *GATA4* | 0/0 | 0/1 | 0/1 | #N/A |
| chr8:11617240 A>T | rs12458 | *GATA4* | 1/1 | 0/1 | 0/1 | #N/A |
| chr17:8024121 C>T | rs1442849 | *HES7* | 1/1 | 1/1 | 0/1 | #N/A |
| chr17:8024204 T>C | rs75711247 | *HES7* | 0/0 | 0/1 | 0/0 | #N/A |
| chr17:8024237 T>C | rs1442850 | *HES7* | 1/1 | 1/1 | 1/1 | #N/A |
| chr17:8024333 T>C | rs182882481 | *HES7* | 1/1 | 0/1 | 0/1 | #N/A |
| chr17:8024758 C>A | rs114049508 | *HES7* | 0/0 | 0/0 | 0/1 | #N/A |
| chr9:102861613 T>G | rs7024375 | *INVS* | 0/1 | 0/1 | 0/1 | #N/A |
| chr9:103063253 A>G | rs190277417 | *INVS* | 0/0 | 0/1 | 0/0 | #N/A |
| chr19:42882829 TA>T | rs3214618 | *MEGF8* | 0/1 | 1/1 | 0/0 | 0.2931 |
| chr10:72191952 A>G | rs2279253 | *NODAL* | 0/1 | 0/1 | 0/0 | #N/A |
| chr10:72191970 C>G | rs2279254 | *NODAL* | 0/1 | 0/1 | 0/0 | #N/A |
| chr10:72195439 T>C | rs1904589 | *NODAL* | 1/1 | 1/1 | 0/1 | 0.6172 |
| chr10:72195576 G>A | rs77151171 | *NODAL* | 0/0 | 0/0 | 0/1 | 0.001387 |
| chr10:72201294 T>G | Novel | *NODAL* | 0/0 | 0/0 | 0/1 | 0.00004976 |
| chr4:88997021 A>AT | Novel | *PKD2* | 0/1 | 0/0 | 1/1 | #N/A |
| chr4:88997102 C>T | rs2728121 | *PKD2* | 0/1 | 0/0 | 1/1 | #N/A |
| chr17:66527262 T>C | Novel | *PRKAR1A* | 0/1 | 0/0 | 0/1 | #N/A |
| chr17:66527402 A>C | rs116996069 | *PRKAR1A* | 0/0 | 0/0 | 0/1 | #N/A |
| chr17:66527834 T>C | rs9925 | *PRKAR1A* | 0/1 | 0/0 | 1/1 | #N/A |
| chr17:66528367 C>T | rs7977 | *PRKAR1A* | 0/1 | 0/0 | 0/1 | #N/A |
| chr17:66528778 C>G | rs6958 | *PRKAR1A* | 0/1 | 1/1 | 0/1 | #N/A |
| chr3:38589666 T>TCCCTCCTTTTTCCTACTCTCTTCTC | rs45592631 | *SCN5A* | 1/1 | 1/1 | 1/1 | #N/A |
| chr3:38590358 C>CT | rs11414422 | *SCN5A* | 0/1 | 0/0 | 0/1 | #N/A |
| chr3:38590470 A>G | Novel | *SCN5A* | 0/1 | 0/1 | 0/0 | #N/A |
| chr3:38590849 G>A | rs4073796 | *SCN5A* | 0/1 | 0/0 | 0/1 | #N/A |
| chr3:38590850 A>T | rs4073797 | *SCN5A* | 0/1 | 0/0 | 0/1 | #N/A |
| chr3:38591059 G>A | rs41310757 | *SCN5A* | 0/1 | 0/0 | 0/1 | #N/A |
| chr3:38591689 T>C | rs7429945 | *SCN5A* | 0/1 | 0/0 | 0/1 | #N/A |
| chr3:38592406 A>G | rs1805126 | *SCN5A* | 0/1 | 0/0 | 0/1 | 0.3879 |
| chr3:38622467 T>C | rs7430407 | *SCN5A* | 1/1 | 1/1 | 1/1 | 0.9127 |
| chr3:38645420 T>C | rs1805124 | *SCN5A* | 0/1 | 0/1 | 0/0 | 0.222 |
| chr3:38674712 T>C | rs6599230 | *SCN5A* | 0/1 | 1/1 | 0/1 | 0.7737 |
| chr18:45359664 T>A | rs8671 | *SMAD2* | 1/1 | 0/1 | 0/1 | #N/A |
| chr18:45360991 A>G | rs1981 | *SMAD2* | 1/1 | 0/1 | 0/1 | #N/A |
| chr18:45361016 C>CTTAT | rs111850625 | *SMAD2* | 1/1 | 1/1 | 1/1 | #N/A |
| chr18:45362150 C>A | Novel | *SMAD2* | 0/0 | 0/1 | 0/0 | #N/A |
| chr18:45362194 T>C | rs1792671 | *SMAD2* | 1/1 | 0/1 | 0/1 | #N/A |
| chr18:45363214 A>T | rs1792666 | *SMAD2* | 1/1 | 0/1 | 0/1 | #N/A |
| chr18:45365396 CA>C | rs5824709 | *SMAD2* | 1/1 | 0/1 | 0/1 | #N/A |
| chr18:45367484 A>G | rs1787187 | *SMAD2* | 1/1 | 1/1 | 1/1 | #N/A |
| chr15:43236612 C>T | rs7178567 | *UBR1* | 1/1 | 1/1 | 1/1 | #N/A |
| chr15:43237203 T>C | rs3803341 | *UBR1* | 1/1 | 1/1 | 1/1 | #N/A |
| chr15:43237572 T>C | rs16957277 | *UBR1* | 0/1 | 0/1 | 1/1 | 0.04805 |
| chr19:55671337 C>T | rs890872 | *DNAAF3* | 1/1 | 1/1 | 1/1 | 1 |
| chr19:55671374 C>T | rs891187 | *DNAAF3* | 0/1 | 0/0 | 0/1 | 0.2268 |
| chr19:55672055 A>G | rs890871 | *DNAAF3* | 0/1 | 0/1 | 0/0 | 0.08592 |
| chr19:55673145 C>T | rs58824375 | *DNAAF3* | 0/1 | 0/1 | 0/0 | 0.1321 |
| chr19:55673164 T>C | rs56726774 | *DNAAF3* | 0/1 | 0/1 | 0/0 | 0.1418 |
| chr19:55673654 T>C | rs3848618 | *DNAAF3* | 0/1 | 1/1 | 0/0 | 0.06602 |

**Supplementary Table S5:** Variation analysis of known candidate of dextrocardia phenotype with homozygous disease model.

| Genomic coordinates | variation status | Gene | Location | II-1 | I-1 | I-2 | TGP MAF | ExAC allele frequency |
| --- | --- | --- | --- | --- | --- | --- | --- | --- |
| **chr17:g.8024333T>C** | **rs182882481** | ***HES7*** | **3'UTR** | **1/1** | **0/1** | **0/1** | **0.001** | #N/A |
| chr8:g.11616338A>C | rs867858 | *GATA4* | 3'UTR | 1/1 | 0/1 | 0/1 | 0.358 | #N/A |
| chr8:g.11616547C>G | rs12825 | *GATA4* | 3'UTR | 1/1 | 0/1 | 0/1 | 0.453 | #N/A |
| chr8:g.11617240A>T | rs12458 | *GATA4* | 3'UTR | 1/1 | 0/1 | 0/1 | 0.392 | #N/A |
| chr18:g.45359664T>A | rs8671 | *SMAD2* | 3'UTR | 1/1 | 0/1 | 0/1 | 0.423 | #N/A |
| chr18:g.45360991A>G | rs1981 | *SMAD2* | 3'UTR | 1/1 | 0/1 | 0/1 | 0.385 | #N/A |
| chr18:g.45362194T>C | rs1792671 | *SMAD2* | 3'UTR | 1/1 | 0/1 | 0/1 | 0.384 | #N/A |
| chr18:g.45363214A>T | rs1792666 | *SMAD2* | 3'UTR | 1/1 | 0/1 | 0/1 | 0.423 | #N/A |
| chr18:g.45365396CA>C | rs5824709 | *SMAD2* | 3'UTR | 1/1 | 0/1 | 0/1 | 0.66 | #N/A |

**Supplementary Table S6:** Compound heterozygous model WES data.

| **Genomic coordinate** | **Gene** | **Effect** | **II-1** | **I-1** | **I-2** | ExAC allele frequency |
| --- | --- | --- | --- | --- | --- | --- |
| chr11: g.6540998 G>T | *DNHD1* | missense | 0/1 | 0/0 | 0/1 | 0.00005698 |
| chr11: g.6585264 C>CCT | *DNHD1* | frameshift | 0/1 | 0/1 | 0/0 | 0.0001998 |
| chr14: g.105223033 G>A | *SIVA1* | missense | 0/1 | 0/1 | 0/0 | 0.00000816 |
| chr14: g.105223055 G>T | *SIVA1* | missense | 0/1 | 0/0 | 0/1 | #N/A |
| chr7: g.151896438 T>A | *KMT2C* | missense | 0/1 | 0/1 | 0/0 | #N/A |
| chr7: g.151917753 G>T | *KMT2C* | missense | 0/1 | 0/0 | 0/1 | #N/A |

**Supplementary Table S7:** The variation found in the homozygous stretch on chr17.

| Genomic coordinates | ID | Gene | effect | ExAC allele frequency |
| --- | --- | --- | --- | --- |
| chr17:g.6942111 G>T | rs33979567 | *SLC16A13* | synonymous | 0.1964 |
| chr17:g.7224921 C>G | rs3809813 | *NEURL4* | missense | 0.1928 |
| chr17:g.7257185 C>T | rs3809830 | *KCTD11* | 3'UTR | #N/A |
| chr17:g.7257669 C>A | rs3809831 | *KCTD11* | 3'UTR | #N/A |
| chr17:g.7257847 GGT>G | rs3840878 | *KCTD11* | 3'UTR | #N/A |
| chr17:g.7360110 T>C | rs2302764 | *CHRNB1* | 3'UTR | #N/A |
| chr17:g.7363055 A>G | rs371972614 | *ZBTB4* | 3'UTR | #N/A |
| chr17:g.7369541 C>T | Novel | *ZBTB4* | missense | 0.002471 |
| chr17:g.7460517 G>C | rs3803798 | *TNFSF12/TNFSF12-TNFSF13* | synonymous | 0.5432 |
| chr17:g.7554536 T>C,G | rs1050528 | *ATP1B2* | 5'UTR | #N/A |
| chr17:g.7554772 C>T | rs1642762 | *ATP1B2* | 5'UTR | #N/A |
| chr17:g.7560294 C>T | rs1050533 | *ATP1B2* | 3'UTR | #N/A |
| chr17:g.7836105 G>A | rs72841443 | *CNTROB* | 5'UTR | #N/A |
| chr17:g.7942434 A>G | rs73972649 | *ALOX15B* | 5'UTR | 0.3069 |
| chr17:g.8024333 T>C | rs182882481 | *HES7* | 3'UTR | #N/A |
| chr17:g.8048216 C>T | Novel | *PER1* | missense | 0.00009358 |
| chr17:g.8062993 G>A | rs75056600 | *VAMP2* | 3'UTR | #N/A |
| chr17:g.8138233 T>G | Novel | *CTC1* | missense | #N/A |
| chr17:g.8222870 C>T | rs79993581 | *ARHGEF15* | missense | 0.02492 |
| chr17:g.8224276 T>C | rs3744647 | *ARHGEF15* | missense | 0.6115 |
| chr17:g.8224670 G>C | rs1045161 | *ARHGEF15* | 3'UTR | #N/A |
| chr17:g.8225768 A>G | rs1045172 | *ARHGEF15* | 3'UTR | #N/A |
| chr17:g.8225787 C>T | rs2430 | *ARHGEF15* | 3'UTR | #N/A |
| chr17:g.8243661 G>A | rs12601097 | *ODF4* | missense | 0.5916 |
| chr17:g.8243785 A>G | rs12936935 | *ODF4* | missense | 0.5896 |
| chr17:g.8272767 G>A | rs2430949 | *KRBA2* | synonymous | 0.1453 |
| chr17:g.8701116 G>A | rs2242374 | *MFSD6L* | synonymous | 0.1367 |
| chr17:g.8702205 C>T | Novel | *MFSD6L* | synonymous | 0.00005714 |
| chr17:g.9674901 C>T | rs114469918 | *DHRS7C* | synonymous | 0.04659 |
| chr17:g.9674922 A>G | rs11654889 | *DHRS7C* | synonymous | 0.07677 |
| chr17:g.9674976 T>C | rs11655932 | *DHRS7C* | synonymous | 0.09058 |

References

1. Bolger, A., Lohse, M. and Usadel, B. Trimmomatic: a flexible trimmer for Illumina sequence data. Bioinformatics, 2014 p.170
2. Lunter G, Goodson M. Stampy: a statistical algorithm for sensitive and fast mapping of Illumina sequence reads. Genome Res 2011;21:936-9
3. McKenna A, Hanna M, Banks E, et al. The Genome Analysis Toolkit: a MapReduce framework for analyzing next-generation DNA sequencing data. Genome Res 2010;20:1297-303.
